# The Relationship between Future Anxiety Due to COVID-19 and Vigilance: The Role of Message Fatigue and Autonomy Satisfaction

**DOI:** 10.3390/ijerph19031062

**Published:** 2022-01-18

**Authors:** Roselyn J. Lee-Won, Inyoung Jang, Hyun-Suk Kim, Sung-Gwan Park

**Affiliations:** 1 School of Communication, The Ohio State University, Columbus, OH 43210, USA; lee-won.1@osu.edu; 2 Department of Communication, Seoul National University, Seoul 08826, Korea; jiy1829@snu.ac.kr (I.J.); hyunsuk.kim@snu.ac.kr (H.-S.K.)

**Keywords:** COVID-19, pandemic, future anxiety, message fatigue, autonomy, vigilance

## Abstract

How does future anxiety caused by the COVID-19 pandemic relate to people’s willingness to remain vigilant and adhere to preventive measures? We examined the mediating role of message fatigue and the moderating role of autonomy satisfaction in the relationship between future anxiety due to COVID-19 and willingness to remain vigilant. A cross-sectional online survey was conducted with adults residing in the United States in June 2021 when numerous U.S. states re-opened following the CDC’s relaxed guidelines for fully vaccinated individuals. Our data showed that message fatigue mediated the relationship between future anxiety due to the pandemic and willingness to remain vigilant. The data further revealed that autonomy satisfaction significantly moderated the mediation. Namely, the role of message fatigue in the indirect relationship between future anxiety and willingness to remain vigilant was significant only among people low to moderate in autonomy satisfaction; its role in the indirect path was not significant for those high in autonomy satisfaction. Notably, independent of the mechanism involving message fatigue, future anxiety was directly and positively associated with willingness to remain vigilant regardless of the levels of autonomy satisfaction. Implications of these findings are discussed in light of psychological and behavioral responses to the current pandemic and policy directions.

## 1. Introduction

“Do not halloo,” as the saying goes, “till you are out of the wood.” One of the most important lessons brought home by the current COVID-19 pandemic is that finding a predictable path through a pandemic is easier said than done. The World Health Organization (WHO) declared the SARS-CoV-2 (COVID-19) virus outbreak “a public health emergency of international concern” on 30 January 2020; less than two months later, on 11 March 2020, WHO declared the COVID-19 situation as a pandemic [1]. Throughout this pandemic, a seeming improvement in the situation (e.g., decreases in positive cases and deaths) led to relaxation of restrictions, which was soon followed by another surge.

It may be stated that the fourth surge in the U.S., largely driven by the Delta variant, was also partly linked to the eased restrictions back in May 2021. On Thursday, 13 May 2021, Dr. Rochelle Walensky—the Director of the U.S. Centers for Disease Control and Prevention (CDC)—announced that mask-wearing and social distancing would no longer be required for fully vaccinated people in the U.S. [2]. While the revised guidelines were welcomed by many, concerns were raised that the guidelines were premature, as most Americans were not fully vaccinated and a viable system for verifying people’s vaccination status was not being implemented [3]. Following these updated CDC guidelines, many states and businesses began lifting mask-wearing mandates and social distancing requirements [4,5]. Then, during the first and the second week of July, the average number of positive COVID-19 cases doubled across the U.S. [6], and by mid-August, COVID-19-related deaths began to rise again [7]. It was not until the end of September that the fourth wave showed some signs of ebbing [8]. The fourth wave in the U.S. poignantly illustrates how a pandemic such as COVID-19 can catch us off-guard again and again when we fail to remain vigilant in the fight against the infectious disease.

The U.S. situation described above highlights the importance of understanding the psychological factors that can influence people’s vigilance in their response to the pandemic. Vigilance in public health crises refers to sustained attention and watchfulness aimed at minimizing risk and enhancing safety [9,10,11]. Willingness to remain vigilant and not to let one’s guard down at the signs of improvement in the fight against the pandemic requires one’s willful intention to sacrifice convenience and adhere to preventive measures for the safety of oneself and others [12]. Given this, identifying factors associated with willingness to remain vigilant may provide important guidelines that can prevent people from being caught off-guard when the pandemic is not completely under control.

To our knowledge, little research has been conducted to identify psychological correlates of vigilance in the prolonged COVID-19 pandemic situation. Most extant research on pandemic response focused on the early phases of the pandemic (e.g., [13,14,15,16]), and evidence has only begun to emerge with respect to how psychological factors such as future anxiety and pandemic-related fatigue are associated with compliance with pandemic measures (e.g., [16]). It may be expected that future anxiety due to COVID-19 can increase people’s motivation to engage in behaviors for preventing the spread of the virus [17]. However, extant literature suggests that future anxiety may induce pandemic-related fatigue, which may, in turn, reduce engagement in protective behaviors [16], and research has yet to examine how future anxiety and pandemic-related fatigue relate to vigilance while the pandemic evolves through multiple waves. It is also important to identify segments of individuals who may be more/less likely to remain vigilant even when the pandemic situation seems to improve but is not completely under control.

In an effort to fill the gap in the extant research on psychological and behavioral responses to the COVID-19 pandemic, the present research investigated the relationship between future anxiety due to the COVID-19 pandemic and willingness to remain vigilant, focusing on a time period when the pandemic-related restrictions were relaxed following multiple major surges—approximately three weeks after the CDC’s announcement of revised mask-wearing and social distancing guidelines. In examining the relationship between future anxiety and willingness to remain vigilant, we tested message fatigue [18,19] as a possible mediator and autonomy satisfaction [20] as a possible moderator. Our approach was informed by psychological reactance theory and self-determination theory. We drew from psychological reactance theory [21,22] to understand the role of message fatigue in the relationship between future anxiety and willingness to remain vigilant, and we explored the possible moderating role of autonomy satisfaction with the self-determination theory framework [20,23,24,25] as our guide.

### 1.1. Future Anxiety, Message Fatigue, and Willingness to Remain Vigilant

In predicting people’s compliance with COVID-19 measures to mitigate the spread of the virus, studies have identified several important variables, and among them are future anxiety and pandemic-related fatigue. Future anxiety refers to a state of feeling uncertainty, fear, and worry induced by the prospect of unfavorable changes in one’s future [26,27]. Although anxiety in general entails perception of the future to some degree, future anxiety concerns a more distant personal future [26]. The COVID-19 pandemic has brought unprecedented disruptions to people’s lives for an extended period of time, resulting in a great amount of uncertainty about the future [28,29], which may increase the anxiety people feel about their future, as has already been indicated in a number of previous studies [17,30,31,32]. Furthermore, it has been demonstrated that high levels of uncertainty and anxiety about the future induced by the pandemic situation increased fatigue [16,33]. Fatigue, as a psychological state of being burned out or lacking motivation [34,35], tends to be experienced during or after extended periods of strenuous activity or stressful events [36,37] and can be exacerbated when people suffer prolonged anxiety because anxiety depletes psychological energy [38] and cognitive resources [39,40]. In exploring the role of future anxiety and pandemic-related fatigue in compliance with pandemic measures, a cross-sectional survey study conducted with Italian adults demonstrated that future anxiety and fatigue serially mediated the relationship between trust in governmental organizations and COVID-19 protective behaviors. Specifically, lower levels of governmental trust led to increased levels of future anxiety, which, in turn, led to increases in the levels of fatigue, and, subsequently, to lower levels of compliance with protective behaviors [16].

Anxiety about the future due to COVID-19 may increase another specific type of fatigue—message fatigue. Message fatigue refers to the state of feeling tired of repeatedly receiving similar messages on a given topic [18]. In consideration of the fact that heightened future anxiety due to COVID-19 has been shown to increase fatigue [30,31], future anxiety could also place people in the state of feeling exhausted and worn out upon being heavily exposed to seemingly repetitive messages concerning COVID-19 [41]. This would be highly likely to happen as the pandemic drags on for an extended period of time and people are exposed to news and public health messages related to COVID-19 over and over. Along this line of reasoning, we hypothesized the following:

**Hypothesis** **1** **(H1).** *Future anxiety due to COVID-19 has a positive association with COVID-19 message fatigue.*

Furthermore, we tested the possible mediating role of message fatigue in the relationship between future anxiety and willingness to remain vigilant. Message fatigue is an aversive motivational state that can drive both passive and active resistance and reactance against the persuasive attempts made by a given message [18,41]. Psychological reactance theory [21] explains why message fatigue contributes to resistance against recommended health behaviors in certain contexts. According to the theory, when an individual’s behavior is restricted by persuasive attempts, the person perceives that his/her freedom is threatened, and this perception of freedom threat motivates the person to resist the persuasive attempts as a way to restore freedom [21,22,42]. Given this, individuals who receive unsolicited and similar health messages that recommend certain health behaviors over an extended period of time may perceive their freedom threatened and exhibit noncompliance as a way to restore freedom [41]. In line with the psychological reactance perspective, a cross-sectional survey study conducted with U.S. adults showed that message fatigue had a positive association with perceived freedom threat toward COVID-19 messages, which, in turn, was positively associated with reactance against COVID-19 preventive measures [13]. Guided by these ideas, we predicted the following:

**Hypothesis** **2** **(H2).** *COVID-19 message fatigue shows a negative association with willingness to remain vigilant.*

**Hypothesis** **3** **(H3).** *The indirect relationship between future anxiety and willingness to remain vigilant through COVID-19 message fatigue is negative.*

**Figure 1:**
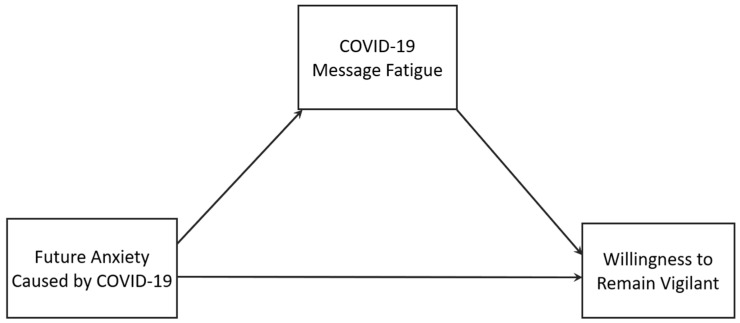
Illustration of the proposed mediation model.

The mediation model to be tested is illustrated in Figure 1.

### 1.2. Autonomy Satisfaction as a Moderator

The link between future anxiety due to COVID-19 and message fatigue may be moderated by autonomy satisfaction as an individual difference trait that varies from person to person, as perception of freedom threat is fundamentally connected to the extent that one perceives his/her autonomy satisfied or thwarted [43,44]. According to self-determination theory [45], which explains how innate psychological needs drive people to exercise their ability to manage and regulate themselves [46], autonomy—the need to actualize the self as the core source of action through freedom [46,47]—is a psychological need that can facilitate behavioral regulation when satisfied and supported [46,48,49].

Autonomy satisfaction, as an individual’s propensity to perceive his/her need for personal volition of actions satisfied [50], has been found as a positive predictor of well-being [51]. In the context of the current COVID-19 pandemic, a study conducted in Serbia found that satisfaction of autonomy, among basic psychological needs, had the greatest influence on well-being [52]. More relevant to the present research, autonomy satisfaction has been identified as an important predictor of effective and lasting health behavior change [48,49]. People are more likely to adopt health behaviors recommended for them when they find their need for autonomy well-supported and satisfied [53]. In a similar vein, autonomous self-regulation as an individual difference variable has been found to shape how people process public health messages [54]. Persuasive health messages encouraging vaccination for health professionals were effective only when the messages were constructed in an autonomy-supportive communication style, and this effect was moderated by individual differences in autonomy-based motivation regulation [55].

**Figure 2:**
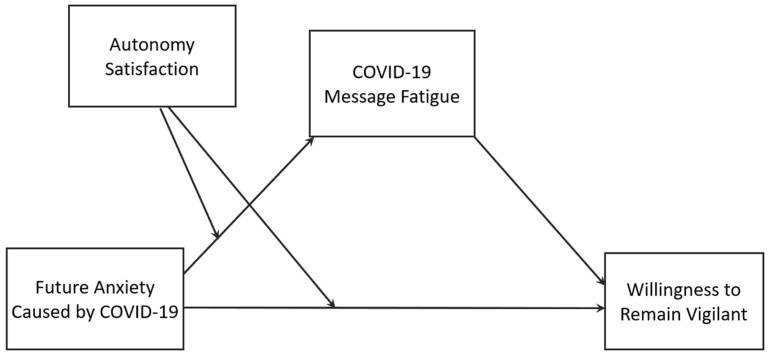
Illustration of the proposed moderated mediation model.

As a propensity that varies from individual to individual, autonomy satisfaction can moderate the path between future anxiety and message fatigue, as people who perceived themselves to be a central target of health promotional messages were more likely to experience message fatigue than those who did not feel targeted by the messages [56,57,58]. Furthermore, given that message fatigue triggered resistance against the behaviors recommended for COVID-19 mitigation [13] and individuals lower in autonomy satisfaction are less likely to engage in self-regulation [59], the indirect path from future anxiety to willingness to remain vigilant via message fatigue can also be moderated by autonomy satisfaction. However, as the specific patterns of moderation may be difficult to predict, we postulated research questions as opposed to hypotheses. First, we asked whether the relationship between future anxiety due to COVID-19 and message fatigue would be moderated by autonomy satisfaction, and, if so, how (*Research Question 1*). Next, we asked whether the mediation hypothesized in H3 would be moderated by autonomy satisfaction, and, if so, how (*Research Question 2*). In addition, we asked whether the direct path between future anxiety and willingness to remain vigilant (i.e., without the involvement of the mediation by message fatigue) would be moderated by autonomy satisfaction (*Research Question 3*). Here, we considered two competing possibilities: (a) In line with the perspectives of psychological reactance theory [21], the direct path might be moderated by autonomy satisfaction, with individuals lower in autonomy satisfaction—perceiving greater freedom threat—exhibiting greater resistance against recommended behavior as a way to restore volition and reduce the anxiety; (b) alternatively, future anxiety might have a positive association with willingness to remain vigilant regardless of autonomy satisfaction, as future anxiety may motivate individuals to engage in protective action for favorable future outcomes [26]. The moderated mediation model incorporating these three research questions is illustrated in Figure 2.

## 2. Materials and Methods

### 2.1. Ethical Considerations

The protocol of this research was determined as exempt from IRB review by the first author’s institution (protocol #2021E0447). Participant recruitment was conducted by Qualtrics, and their recruitment approach did not involve any form of coercion. Those who were invited to participate in the survey could simply refuse to click on the invitation links sent out by the panel managers. In addition, the incentives offered in the form of reward points, which could be redeemed for rewards of the participant’s choice, were moderate. Because the compensation procedure was managed by the panel providers partnering with Qualtrics and because the incentives were prorated, the exact number of reward points was not predetermined by the research team. All participants provided their consent prior to participating in this online survey.

### 2.2. Participants

All of our participants were recruited through the service provided by Qualtrics, which partners with a number of online survey firms that manage opt-in panels for online research. The opt-in panel service provided by Qualtrics has been successfully utilized in the recruitment of participants for online research [60].

Our inclusion criteria were (a) being 18–65 years of age and (b) living in the U.S. at the time of data collection. In response to the email invitations sent out by the online panel managers, 1151 individuals clicked the survey invitation link, and a total of 487 individuals completed the survey, with a completion rate of 42.3%. Of the 487 participants, 47.6% (*n* = 232) were female. More information on the sample characteristics is provided in the Section 2.4.5 Control Variables below.

### 2.3. Procedure

Our data were collected in June 2021. At the time of our data collection, over half of the eligible individuals in the U.S. were fully vaccinated, and states began to scale back their daily tracking of COVID-19 cases [61], following the CDC’s announcement of the relaxed mask-wearing and social distancing guidelines for fully vaccinated individuals in May 2021.

### 2.4. Measures

#### 2.4.1. Future Anxiety Due to COVID-19 (“Dark Future” Scale)

Anxiety about the future caused by the COVID-19 pandemic situation was measured with five items adapted from the Dark Future scale [27]. As the original items focused more on future anxiety as an individual difference variable, they were slightly rephrased to highlight the state of future anxiety felt in the context of the current COVID-19 pandemic. The instructions for participants read: “The statements below concern your attitude towards the future. Select the number that most accurately defines your point of view. There are no ‘right’ or ‘wrong’ answers.” The items were: “I am afraid that the problems caused by the COVID-19 pandemic that trouble me now will continue for a long time”; “I am terrified by the thought that I might suffer long-term crises or difficulties due to the COVID-19 pandemic”; “I am afraid that in the future my life will change for the worse due to the COVID-19 pandemic”; “I am afraid that the impact of the COVID-19 pandemic on the economy will threaten my future”; and “I am disturbed by the thought that the COVID-19 pandemic will prevent me from realizing my goals.” Participants rated the statements on a 7-point scale, ranging from 0 (*decidedly false*) to 6 (*decidedly true*). The ratings were averaged (Cronbach α = 0.94; *M* = 2.65; *SD* = 1.63).

#### 2.4.2. Message Fatigue

Following Ball and Wozniak [13], we measured COVID-19 message fatigue by adapting the four-item *Exhaustion* subscale of the Message Fatigue scale [18]. On a 7-point scale (1 = *strongly disagree* vs. 7 = *strongly agree*), participants were asked to rate their agreement with the following four statements: “I am burned out from hearing that COVID-19 is a serious problem”; “I am sick of hearing about problems associated with COVID-19”; “I am tired of hearing about the importance of social distancing and mask wearing to prevent the spread of COVID-19”; and “Health messages about COVID-19 make me want to sigh.” The ratings were averaged (α = 0.93; *M* = 4.20; *SD* = 1.78).

#### 2.4.3. Willingness to Remain Vigilant

Willingness to remain vigilant was measured with four items adapted from the Willingness-to-Sacrifice scale [62]. Participants were asked to rate the following statements on a 7-point scale (1 = *strongly disagree* vs. 7 = *strongly agree*): “I am willing to sacrifice convenience to help prevent the spread of COVID-19”; “I am willing to adhere to mask wearing in public settings even if mask wearing is not required”; “I am willing to adhere to social distancing in public settings even if restrictions are completely lifted”; and “I am willing to be mindful of others who may be vulnerable to COVID-19.” The ratings were averaged (α = 0.92; *M* = 5.30; *SD* = 1.55).

#### 2.4.4. Autonomy Satisfaction

In order to measure individual differences in autonomy satisfaction in the context of the COVID-19 pandemic, we adapted items from the Autonomy Satisfaction subscale of the Basic Psychological Needs scale [63]. The question prompt read: “Below, we ask you about the kind of experiences you actually have in your life. Please indicate the degree to which the statement is true for you at this point in your life.” Following the prompt, participants rated four statements on a 5-point scale (1 = *not true at all* vs. 5 = *completely true*): “Despite the current COVID-19 pandemic situation, I still feel a sense of choice and freedom in the things I undertake”; “Despite the current COVID-19 pandemic situation, I still feel that my decisions reflect what I really want”; “Despite the current COVID-19 pandemic situation, I still feel my choices express who I really am”; and “Despite the current COVID-19 pandemic situation, I still feel I am doing what really interests me.” The ratings were averaged (α = 0.88; *M* = 3.90; *SD* = 0.88).

#### 2.4.5. Control Variables

We controlled for potential confounders, which included demographic variables (age, gender, education, household income, and race), political orientation, loss of family/friends due to COVID-19, vaccination status, and relaxation of COVID-19 restrictions in the state in which participants resided at the time of this study.

Age (in years) was asked as an open-ended question (*M* = 43.10; *SD* = 13.81). As for gender (0 = *male* vs. 1 = *female*; *M* = 0.48; *SD* = 0.50), 47.6% (*n* = 232) were female. As for race, 78.9% of the participants were White, 7.4% were Black or African American, 0.8% were American Indian or Alaskan Native, 5.1% were Hispanic, Latino/a, or Spanish origin, 6.6% were Asian/Asian American, 0.2% were Native Hawaiian or other Pacific Islander, and 1.0% responded “Other”; the race variable was dichotomized for the analysis (0 = White vs. 1 = non-White; *M* = 0.21; *SD* = 0.41). Education was measured with five response categories, and their values ranged from 1 to 5 (*M* = 3.32; *SD* = 1.00): 1 = *less than a high school degree* (3.5%), 2 = *high school degree or GED* (18.1%), 3 = *some college or technical school* (32.0%), 4 = *college degree* (35.9%), and 5 = *more than college degree* (10.5%). Household income was measured with five response categories, with values ranging from 1 to 5 (*M* = 2.59; *SD* = 1.27): 1 = *less than USD 20,000* (22.6%), 2 = *USD 20,000 to USD 49,999* (30.0%), 3 = *USD 50,000 to USD 89,999* (25.5%), 4 = *USD 90,000 to USD 119,999* (10.1%), and 5 = *USD 120,000 or above* (11.9%).

The question on loss of family/friends due to COVID-19 read, “To the best of your knowledge, how many of your family members, close relatives, and friends passed away from COVID-19?” Participants’ responses were dichotomized (0 = *none lost to COVID-19* vs. 1 = *one or more people lost to COVID-19*); 106 individuals (21.8%) responded that they had lost one or more family members or friends to COVID-19 (*M* = 0.22; *SD* = 0.41). Political orientation was measured on a 5-point scale, 1 = *very conservative* vs. 5 = *very liberal* (*M* = 2.99; *SD* = 1.23). The vaccination status question read, “Have you received at least one dose of a COVID-19 vaccine?” (0 = *no* vs. 1 = *yes*); 277 participants (56.9%) reported that they received at least one dose of a COVID-19 vaccine (*M* = 0.57; *SD* = 0.50). Finally, the degree to which one’s state relaxed its COVID-19 restrictions was assessed with four questions with two response options (0 = *no* vs. 1 = *yes*): “Do most indoor businesses operate without capacity limits in your state?”; “Do most businesses no longer require mask wearing in your state?”; “Is mask wearing required in indoor public settings in your state?” (reversed); and “Does your state still mandate businesses to observe social distancing rules?” (reversed). Scores were added, with higher scores indicating greater degrees of relaxed COVID-19 restrictions in the participants’ states of residence (*M* = 2.11; *SD* = 1.30).

## 3. Results

### 3.1. Overview of the Analysis

The bivariate correlation coefficients of all measured variables are provided in the supplementary document available online (Table S1). Based on Ordinary Least Squares (OLS) regression, we estimated a path model to address (a) the hypotheses on indirect relationships (H1–H3) and (b) the research questions on moderation (RQ1 and RQ3) and moderated mediation (RQ2). For statistical inference in mediation analysis and moderated mediation analysis, we used percentile bootstrap confidence intervals (CIs) with 10,000 bootstrap samples. All analyses were conducted using the PROCESS macro [64]. Our mediation hypotheses (H1–H3) were tested with Model 4, and the research questions on moderation and moderated mediation (RQ1–RQ3) were addressed with Model 8 [65]; in the moderation and moderated mediation analysis, future anxiety due to COVID-19 and autonomy satisfaction were mean-centered.

### 3.2. Mediation Analysis

**Figure 3:**
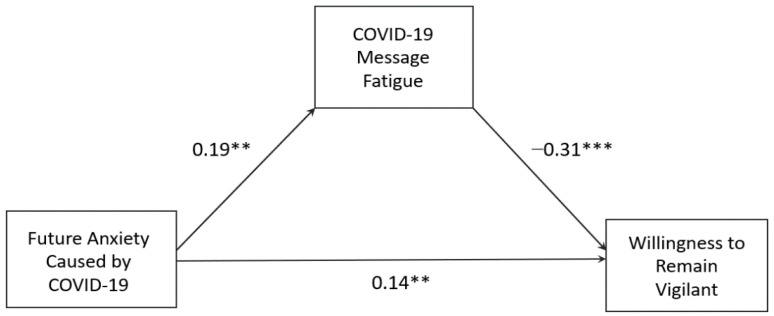
The mediation model predicting willingness to remain vigilant. The paths show unstandardized coefficients. ** *p* < 0.01. *** *p* < 0.001.

H1 predicted that anxiety about the future caused by COVID-19 would have a positive association with COVID-19 message fatigue. Our data supported this hypothesis, *b* = 0.19, *p* = .001, 95% CI [0.10, 0.28]. H2 predicted that COVID-19 message fatigue would show a negative association with willingness to remain vigilant. This hypothesis was supported by the data, *b* = −0.31, *p* < .001, 95% CI [−0.38, −0.24]. Finally, H3 predicted that the indirect relationship between future anxiety and willingness to remain vigilant through COVID-19 message fatigue would be negative. Our data supported this mediation hypothesis. The coefficient for the indirect path was significant and negative, point estimate = −0.06, 95% bootstrap CI [−0.09, −0.02]. Notably, independent of this mechanism involving COVID-19 message fatigue as the mediator, the direct path from future anxiety to willingness to remain vigilant was significantly positive, *b* = 0.14, 95% CI [0.07, 0.21], *p* = .001. Figure 3 illustrates these results.

### 3.3. Moderation and Moderated Mediation Analysis

**Figure 4:**
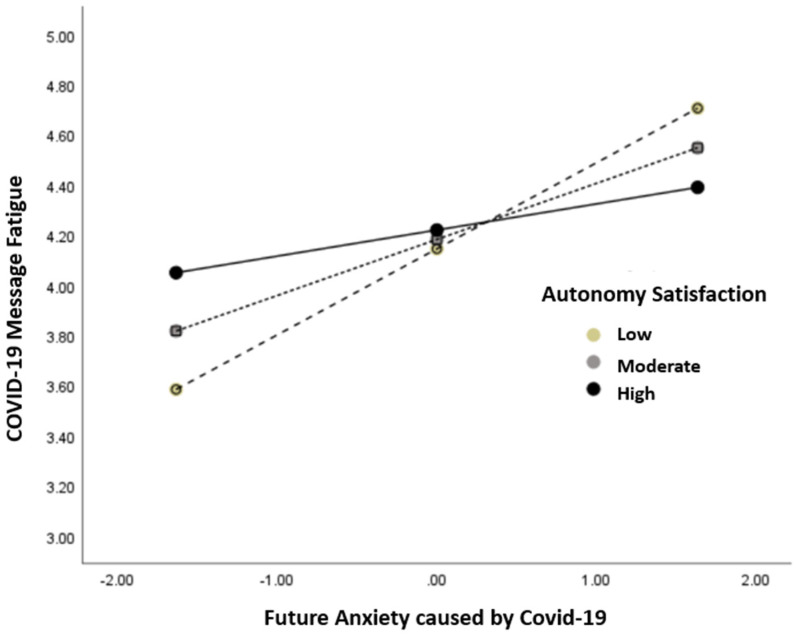
Future Anxiety and Message Fatigue: Moderation by Autonomy Satisfaction.

The research questions were addressed with OLS regression based on Model 8 of the PROCESS macro (Table 1). RQ1 asked whether and how the relationship between future anxiety due to COVID-19 and COVID-19 message fatigue would be moderated by autonomy satisfaction. The analysis revealed a negative interaction between future anxiety and autonomy satisfaction predicting COVID-19 message fatigue, *b* = −0.14, 95% CI [−0.23, −0.04], *p* = .004, such that autonomy satisfaction weakened the association between future anxiety and message fatigue: *b* = 0.34, 95% CI [0.21, 0.48], *p* < .001 for respondents low (*M −* 1*SD*) in autonomy satisfaction (–0.88); *b* = 0.22, 95% CI [0.13, 0.32], *p* < .001 for those moderate (*M*) in autonomy satisfaction (0); *b* = 0.10, 95% CI [−0.004, 0.21], *p* = .06 for those high (*M* + 1*SD*) in autonomy satisfaction (0.88). This interaction pattern is illustrated in Figure 4.

RQ2 asked whether and how the mediation hypothesized in H3 would be moderated by autonomy satisfaction, with moderation of the mechanism proposed as driven by the first stage of the mediation process involving the path from future anxiety to message fatigue. Our analysis revealed that the index of moderated mediation (index = 0.04) was significantly different from zero, 95% bootstrap CI [0.01, 0.08], indicating that the size of the indirect relationship predicted in H3 (future anxiety due to COVID-19 → COVID-19 message fatigue → willingness to remain vigilant) was significantly affected by autonomy satisfaction. This significant moderated mediation was further probed (Table 2). The mediation predicted in H3 was significant among those moderate (*M*) in autonomy satisfaction (0) and low (*M* − 1*SD*) in autonomy satisfaction (–0.88); on the other hand, the mediation was not significant among those high (*M* − 1*SD*) in autonomy satisfaction (0.88).

We also looked at the direct association between future anxiety due to COVID-19 and willingness to remain vigilant, independent of the aforementioned mechanism involving COVID-19 message fatigue (RQ3). As indicated by the interaction term shown in Table 1, the direct relationship was not significantly moderated by autonomy satisfaction. Rather, as shown in Table 2, the coefficients for the direct paths from future anxiety to willingness to remain vigilant were significant and positive across the three levels of autonomy satisfaction (i.e., “low” to “high”).

## 4. Discussion

### 4.1. Implications of the Key Findings

Our study examined psychological and behavioral responses to the COVID-19 pandemic between the third and the fourth wave in the U.S. context. Specifically, we investigated how future anxiety caused by the pandemic situations was associated with willingness to remain vigilant when people might be inclined to relax precautions as COVID-19 restrictions eased. In so doing, we examined the mediating role of message fatigue and the moderating role of autonomy satisfaction.

We found that the relationship between future anxiety caused by COVID-19 and willingness to remain vigilant was mediated by COVID-19 message fatigue. Specifically, future anxiety was positively related to message fatigue, which, in turn, was negatively associated with willingness to remain vigilant to protect the self and others from the disease. Interestingly, independent of the mechanism involving COVID-19 message fatigue, greater future anxiety was associated with greater willingness to remain vigilant.

Moreover, we found that autonomy satisfaction moderated the path between future anxiety and message fatigue. Specifically, for those low and moderate in autonomy satisfaction, future anxiety had a positive association with message fatigue; however, for those high in autonomy satisfaction, the relationship between future anxiety and message fatigue was only marginally significant. Additionally, the indirect path between future anxiety and willingness to remain vigilant through message fatigue was also moderated by autonomy satisfaction. Namely, the indirect relationship was significant among people low to moderate in autonomy satisfaction but not among those high in autonomy satisfaction. Notably, independent of the mechanism involving message fatigue, the direct relationship between future anxiety and willingness to remain vigilant was positive and not moderated by autonomy satisfaction. These results highlighted that message fatigue (associated with future anxiety), being contingent upon autonomy satisfaction as an individual difference variable, may play a potentially detrimental role, particularly when people are likely to become weary of the prolonged pandemic.

The findings of our study contribute to the literature on pandemic responses in meaningful ways. First, the current findings provide insight into how people cope with future anxiety caused by the pandemic situations and how message fatigue and autonomy satisfaction may come into play in the coping process, particularly in light of the theory of stress and coping [66,67]. According to this perspective, which highlights how cognitive appraisal of stressors initiates the coping process, there are two primary routes of coping: emotion-focused coping and problem-focused coping [67]. Emotion-focused coping, which refers to the coping response governed by the regulation of emotions triggered by the cognitive appraisal of a stressor, has an internal focus and solely aims at mitigating the undesirable emotions induced by stress and anxiety. On the other hand, problem-focused coping, which refers to the coping response centered around the effort to tackle the problems directly associated with the stressor, has an external focus and aims at changing the unfavorable situation that causes stress and anxiety by behaviorally acting on the problem itself [67,68,69]. Our findings suggest that coping with future anxiety caused by COVID-19 may also follow either of these two routes of coping, with message fatigue and autonomy satisfaction determining which of the two routes to be taken. As indicated by the results on the direct relationships between future anxiety and willingness to remain vigilant, people may engage in problem-focused coping when dealing with future anxiety and thereby exhibit willingness to act upon the problem—the COVID-19 pandemic—when message fatigue does not intervene. However, when message fatigue intervenes, future anxiety may be associated with lower motivation to remain vigilant, particularly among those who are lower in autonomy satisfaction. These individuals may engage in emotion-focused coping as opposed to problem-focused coping.

Our findings aligned with prior research on message fatigue and resistance against persuasive public health messages [13,41,58,70]. Although achieving a high level of exposure is key to successful public health communication campaigns [71], massive messaging also has the potential to invite resistance to its persuasive attempt by repeatedly exposing people to similar health information and persuasive arguments, thereby increasing message fatigue [18]. This might be particularly true in the era of the COVID-19 pandemic in which an unprecedent level of public health messaging is undertaken around the globe, disseminating important but similar messages [70,72]. Furthermore, our research also contributes to the literature by indicating that future anxiety, particularly in a prolonged pandemic situation, may serve as a possible predictor of message fatigue.

On a positive note, our test of the moderated mediation model highlighted that autonomy satisfaction, as one of the basic psychological needs [52], may play a protective role by suppressing the relationship between future anxiety and message fatigue. By highlighting the role of autonomy satisfaction as an important individual difference variable, this finding will guide us to identify individuals who may be more vulnerable to experiencing message fatigue (i.e., those who are lower in autonomy satisfaction). Another noteworthy implication of this finding with respect to autonomy satisfaction is that in order for people to constructively cope with future anxiety caused by the prolonged pandemic situation, people’s need for autonomy as a basic psychological need should remain satisfied and well-supported [52].

From a practical standpoint with respect to public health message design concerning COVID-19 and future pandemics, our findings can inspire the development of responses to the current and future pandemic situations. First, our results suggest that public health messages should focus on how to reduce future anxiety by addressing the probability and severity of potential harms related to COVID-19 [73], particularly when reaching out to target individuals low in autonomy satisfaction.

Second, it will be important to help people maintain optimal levels of autonomy satisfaction while they attempt to cope with future anxiety caused by prolonged pandemic situations. Governmental and public health institutions should design and frame preventive measures and related messages in autonomy-supporting ways [52]. Also, special care should be taken to employ message features to mitigate perceived threat to freedom and uplift autonomy satisfaction, such as the use of narratives and choice-enhancing language [42]. Controlling messages that may trigger freedom threat [74] or messages that evoke negative emotions such as guilt or shame may backfire [75], leading to increases in message fatigue and non-compliance with preventive measures [52].

### 4.2. Limitations and Future Directions

Our work has several limitations that should be addressed. First, the correlational nature of our data, collected via a cross-sectional survey, does not allow for causal interpretation of the observed relationships among future anxiety, message fatigue, and willingness to remain vigilant. Specifically, we cannot conclusively rule out the possibility that reverse causation (e.g., intention to remain vigilant lowers message fatigue) and spuriousness (e.g., the observed negative relationship between message fatigue and intention to remain vigilant) are explained by health interests, such that individuals highly interested in health issues are less likely to experience message fatigue and more willing to stay vigilant. Future research should employ a longitudinal survey or experimental design to reduce uncertainty about causality concerning the relationships observed in the current study.

While our study sheds light on how future anxiety induced by the pandemic and autonomy satisfaction jointly influence message fatigue and thereby shape willingness to remain vigilant, the sample was restricted to people residing in the U.S. Future research should test the mediation and the moderated mediation models proposed in this research in other countries and examine the extent to which the current findings can generalize to different societies and cultures.

Additionally, future studies identifying the individual- and message-level antecedents of future anxiety and autonomy satisfaction can illuminate a more comprehensive process—part of which has been illustrated in this study—and ultimately guide the efforts to design effective message interventions to prevent the spread of COVID-19 and future pandemics. Future research should also investigate how information dynamics can affect future anxiety, message fatigue, and willingness to remain vigilant in pandemic situations. The COVID-19 information environment has witnessed rapid and widespread dissemination of misinformation [76] and conspiracy theories [77] as well as politicization of health-protective behaviors such as mask-wearing [78,79]. Being placed in an environment heavily dominated by confusing and conflicting information, the public may be rendered highly vulnerable to future anxiety and message fatigue [80], which could negatively contribute to willingness to remain vigilant.

## 5. Conclusions

We have already witnessed multiple waves of positive cases and deaths throughout the current COVID-19 pandemic, and it is important to understand what predicts people’s (un)willingness to remain vigilant to protect the self and others from the disease. Specifically, understanding psychological and behavioral responses to prolonged pandemic situations that heighten uncertainty and anxiety can provide important insight into effectively encouraging the public to avoid being caught off-guard by not relaxing precautions before the pandemic is completely under control. Guided by psychological reactance theory and self-determination theory, we investigated the relationship between future anxiety and willingness to remain vigilant with a sample of U.S. adults; in doing so, we examined the mediating role of message fatigue and the moderating role of autonomy satisfaction. Despite limitations due to the cross-sectional nature of the data, our findings shed light on the role of message fatigue and autonomy satisfaction in how people appraise anxiety-provoking pandemic situations and regulate their behavior accordingly. Our research also contributes to the public health scholarship by identifying individuals who may be more vulnerable to experiencing message fatigue and thereby rendered less willing to remain vigilant in prolonged pandemic situations. We hope that the present research will stimulate further research on the socio-psychological factors that influence vigilance in pandemic response.

## Figures and Tables

**Table 1:** OLS regression results based on PROCESS Model 8.

	Consequent					
	M (Message Fatigue)			Y (Vigilance)		
Antecedent	Coeff.	*SE*	*p*	Coeff.	*SE*	*p*
Constant	6.08	0.46	<.001	5.99	0.41	<.001
X (Future Anxiety)	0.22	0.05	<.001	0.16	0.04	<.001
M (Message Fatigue)	--	--	--	−0.31	0.03	<.001
W (Autonomy Satisfaction)	0.04	0.08	.611	0.38	0.06	<.001
X× W	−0.14	0.05	.004	0.006	0.04	.878
Age	−0.01	0.005	.186	−0.003	0.004	.424
Gender	0.02	0.15	.880	0.28	0.11	.012
Education	−0.07	0.08	.411	−0.06	0.06	.383
Income	−0.003	0.06	.958	0.16	0.05	.001
Race	−0.28	0.18	.128	0.15	0.14	.294
Political Orientation	−0.49	0.06	<.001	0.18	0.05	.001
COVID death	−0.01	0.18	.965	−0.02	0.14	.869
Vaccination	−0.49	0.16	.002	0.51	0.12	<.001
Eased Restrictions	0.21	0.06	.0002	−0.21	0.04	<.001
	$R^{2}$ = 0.22			$R^{2}$ = 0.39		
Model Summary	F(12,474) = 11.20, *p* < .001			F(13,473) = 23.45, *p* < .001		

Note. Future anxiety and autonomy satisfaction were mean-centered.

**Table 2:** Conditional direct and indirect relationships.

	Indirect Path		Direct Path		
Autonomy Satisfaction	Point Estimate	95% Bootstrap CI	*b*	95% CI	*p*
Low (*M* − 1*SD*)	− **0.11**	**−0.16 to −0.06**	**0.15**	**0.04 to 0.26**	**.006**
Moderate (*M*)	− **0.07**	**−0.11 to −0.03**	**0.16**	**0.08 to 0.23**	**<.001**
High (*M* + 1*SD*)	−0.03	−0.08 to 0.01	**0.16**	**0.08 to 0.25**	**.0002**

Note. Autonomy satisfaction was mean-centered. Statistically significant values are presented in boldface.

## Data Availability

The de-identified data presented in this study will be made available from the authors upon reasonable request.

## Supplementary Materials

The following are available online at https://www.mdpi.com/article/10.3390/ijerph19031062/s1, Table S1: Bivariate Correlations among Measured Variables.

## References

[1] World Health Organization Rolling Updates on Coronavirus Disease (COVID-19). https://www.who.int/emergencies/diseases/novel-coronavirus-2019/events-as-they-happen.

[2] Wamsley L. (2021). Fully vaccinated people can stop wearing masks indoors and outdoors, CDC says. National Public Radio.

[3] Stanley-Becker I., Guarino B., Sellers F.S., Cha A.E., Sun L.H. (2021). CDC’s mask guidance spurs confusion and criticism, as well as celebration. The Washington Post.

[4] Peeples L. (2021). What the science says about lifting mask mandates. Nature.

[5] Yamey G., Chapple-McGruder T. (2021). The CDC’s abrupt change to mask guidelines puts people at risk. Time.

[6] Weintraub K. (2021). The fourth wave of COVID-19 cases is here. Will we escape the UK’s fate? It’s too soon to know. USA Today.

[7] Wilson C. (2021). Driven by the delta variant, the fourth wave of COVID-19 in the U.S. Could be worse than the third. In some states, it already is. Time.

[8] Martínez A., Aubrey A. (2021). Latest data on COVID-19 infections show cases are dropping. National Public Radio.

[9] Weir L., Mykhalovskiy E., Bashford A. (2007). The geopolitics of global public health surveillance in the twenty-first century. Medicine at the Border.

[10] Ilesanmi O.S., Bello A.E., Afolabi A.A. (2020). COVID-19 pandemic response fatigue in Africa: Causes, consequences, and counter-measures. Pan. Afr. Med. J..

[11] Lau B.H.P., Chan C.L.W., Ng S.-M. (2021). Resilience of Hong Kong people in the COVID-19 pandemic: Lessons learned from a survey at the peak of the pandemic in Spring 2020. Asia Pac. J. Soc. Work Dev..

[12] Liu J.H.-F., Leong C.-H., Huang S.-Y., Chen S.X., Choi H.-S., Yamaguchi S., Lee I.-C., Inoue Y. (2020). Pandemic response: Vigilance, civic responsibility critical to East Asia’s success. East. Asia Forum Q..

[13] Ball H., Wozniak T.R. (2021). Why do some Americans resist COVID-19 prevention behavior? An analysis of issue importance, message fatigue, and reactance regarding COVID-19 messaging. Health Commun..

[14] Dillard J.P., Tian X., Cruz S.M., Smith R.A., Shen L. (2021). Persuasive messages, social norms, and reactance: A study of masking behavior during a COVID-19 campus health campaign. Health Commun..

[15] Sobol M., Blachnio A., Przepiórka A. (2020). Time of pandemic: Temporal perspectives related to compliance with public health regulations concerning the COVID-19 pandemic. Soc. Sci. Med..

[16] Scandurra C., Bochicchio V., Dolce P., Valerio P., Muzii B., Maldonato N.M. (2021). Why people were less compliant with public health regulations during the second wave of the Covid-19 outbreak: The role of trust in governmental organizations, future anxiety, fatigue, and Covid-19 risk perception. Curr. Psychol..

[17] Duplaga M., Grysztar M. (2021). The association between future anxiety, health literacy and the perception of the COVID-19 pandemic: A cross-sectional study. Healthcare.

[18] So J., Kim S., Cohen H. (2017). Message fatigue: Conceptual definition, operationalization, and correlates. Commun. Monogr..

[19] Calder B.J., Sternthal B. (1980). Television commercial wearout: An information processing view. J. Mark. Res..

[20] Ryan R.M., Deci E.L. (2006). Self-regulation and the problem of human autonomy: Does psychology need choice, self-determination, and will?. J. Personal..

[21] Brehm J.W. (1966). A Theory of Psychological Reactance.

[22] Rosenberg B.D., Siegel J.T. (2018). A 50-year review of psychological reactance theory: Do not read this article. Motiv. Sci..

[23] Ryan R.M., Deci E.L., Greenberg J., Koole S.L., Pyszczynski T. (2004). Autonomy is no illusion: Self-determination theory and the empirical study of authenticity, awareness, and will. Handbook of Experimental Existential Psychology.

[24] Niemiec C.P., Ryan R.M., Deci E.L., Hoyle R.H. (2010). Self-determination theory and the relation of autonomy to self-regulatory processes and personality development. Handbook of Personality and Self-Regulation.

[25] Niemiec C.P., Ryan R.M., Brown K.W., Wayment H.A., Bauer J.J. (2008). The role of awareness and autonomy in quieting the ego: A self-determination theory perspective. Transcending Self-Interest: Psychological Explorations of the Quiet Ego.

[26] Zaleski Z. (1996). Future Anxiety: Concept, measurement, and preliminary research. Personal. Individ. Differ..

[27] Zaleski Z., Sobol-Kwapinska M., Przepiorka A., Meisner M. (2019). Development and validation of the Dark Future scale. Time Soc..

[28] Rettie H., Daniels J. (2021). Coping and tolerance of uncertainty: Predictors and mediators of mental health during the COVID-19 pandemic. Am. Psychol..

[29] Paredes M.R., Apaolaza V., Fernandez-Robin C., Hartmann P., Yañez-Martinez D. (2021). The impact of the COVID-19 pandemic on subjective mental well-being: The interplay of perceived threat, future anxiety and resilience. Personal. Individ. Differ..

[30] Giallonardo V., Sampogna G., Del Vecchio V., Luciano M., Albert U., Carmassi C., Carrà G., Cirulli F., Dell’Osso B., Nanni M.G. (2020). The impact of quarantine and physical distancing following COVID-19 on mental health: Study protocol of a multicentric italian population trial. Front. Psychiatry.

[31] Usher K., Durkin J., Bhullar N. (2020). The COVID-19 pandemic and mental health impacts. Int. J. Ment. Health Nurs..

[32] Dodd R.H., Dadaczynski K., Okan O., McCaffery K.J., Pickles K. (2021). Psychological wellbeing and academic experience of university students in Australia during COVID-19. Int. J. Environ. Res. Public Health.

[33] Nitschke J.P., Forbes P.A.G., Ali N., Cutler J., Apps M.A.J., Lockwood P.L., Lamm C. (2021). Resilience during uncertainty? Greater social connectedness during COVID-19 lockdown is associated with reduced distress and fatigue. Br. J. Health Psychol..

[34] Cella D., Peterman A., Passik S., Jacobsen P., Breitbart W. (1998). Progress toward guidelines for the management of fatigue. Oncology.

[35] Smets E.M.A., Garssen B., Bonke B., De Haes J.C.J.M. (1995). The multidimensional Fatigue Inventory (MFI) psychometric qualities of an instrument to assess fatigue. J. Psychosom. Res..

[36] Boksem M.A.S., Tops M. (2008). Mental fatigue: Costs and benefits. Brain Res. Rev..

[37] Voci A., Veneziani C.A., Metta M. (2016). Affective organizational commitment and dispositional mindfulness as correlates of burnout in health care professionals. J. Workplace Behav. Health.

[38] Minahan J., Schultz J.J. (2014). Interventions can salve unseen anxiety barriers. Phi Delta Kappan.

[39] Moser J.S., Becker M.W., Moran T.P. (2012). Enhanced attentional capture in trait anxiety. Emotion.

[40] Beaudreau S.A., O’Hara R. (2008). Late-life anxiety and cognitive impairment: A review. Am. J. Geriatr. Psychiatry.

[41] Kim S., So J. (2018). How message fatigue toward health messages leads to ineffective persuasive outcomes: Examining the mediating roles of reactance and inattention. J. Health Commun..

[42] Reynolds-Tylus T. (2019). Psychological reactance and persuasive health communication: A review of the literature. Front. Commun..

[43] Mühlberger C., Jonas E., Sassenberg K., Vliek M.L.W. (2019). Reactance theory. Social Psychology in Action: Evidence-Based Interventions from Theory to Practice.

[44] Bessarabova E., Miller C.H., Russell J. (2017). A further exploration of the effects of restoration postscripts on reactance. West. J. Commun..

[45] Deci E.L., Eghrari H., Patrick B.C., Leone D.R. (1994). Facilitating internalization: The self-determination theory perspective. J. Personal..

[46] Ryan R.M., Deci E.L. (2000). Self-determination theory and the facilitation of intrinsic motivation, social development, and well-being. Am. Psychol..

[47] Skinner E., Edge K., Deci E.L., Ryan R.M. (2002). Self-determination, coping, and development. Handbook of Self-Determination Research.

[48] Ng J.Y.Y., Ntoumanis N., Thøgersen-Ntoumani C., Deci E.L., Ryan R.M., Duda J.L., Williams G.C. (2012). Self-determination theory applied to health contexts: A meta-analysis. Perspect. Psychol. Sci..

[49] Williams G.C., Cox E.M., Kouides R., Deci E.L. (1999). Presenting the facts about smoking to adolescents: Effects of an autonomy-supportive style. Arch. Pediatrics Adolesc. Med..

[50] Coterón J., Franco E., Ocete C., Pérez-Tejero J. (2020). tTeachers’ psychological needs satisfaction and thwarting: Can they explain students’ behavioural engagement in physical education? A multi-level analysis. Int. J. Environ. Res. Public Health.

[51] Tang M., Wang D., Guerrien A. (2020). A systematic review and meta-analysis on basic psychological need satisfaction, motivation, and well-being in later life: Contributions of self-determination theory. PsyCh J..

[52] Šakan D., Žuljević D., Rokvić N. (2020). The role of basic psychological needs in well-being during the COVID-19 outbreak: A self-determination theory perspective. Front. Public Health.

[53] Gillison F.B., Rouse P., Standage M., Sebire S.J., Ryan R.M. (2019). A meta-analysis of techniques to promote motivation for health behaviour change from a self-determination theory perspective. Health Psychol. Rev..

[54] Resnicow K., Zhou Y., Hawley S., Jimbo M., Ruffin M.T., Davis R.E., Shires D., Lafata J.E. (2014). Communication preference moderates the effect of a tailored intervention to increase colorectal cancer screening among African Americans. Patient Educ. Couns..

[55] Moon K., Riege A., Gourdon-Kanhukamwe A., Vallée-Tourangeau G. (2021). The moderating effect of autonomy on promotional health messages encouraging healthcare professionals’ to get the influenza vaccine. J. Exp. Psychol. Appl..

[56] Rowniak S. (2009). Safe sex fatigue, treatment optimism, and serosorting: New challenges to HIV prevention among men who have sex with men. J. Assoc. Nurses AIDS Care.

[57] Stockman J.K., Schwarcz S.K., Butler L.M., de Jong B., Chen S.Y., Delgado V., McFarland W. (2004). HIV prevention fatigue among high-risk populations in San Francisco. JAIDS J. Acquir. Immune Defic. Syndr..

[58] So J., Alam N. (2019). Predictors and effects of anti-obesity message fatigue: A thought-listing analysis. Health Commun..

[59] Williams G.C., Grow V.M., Freedman Z.R., Ryan R.M., Deci E.L. (1996). Motivational predictors of weight loss and weight-loss maintenance. J. Personal. Soc. Psychol..

[60] Boas T.C., Christenson D.P., Glick D.M. (2020). Recruiting large online samples in the United States and India: Facebook, Mechanical Turk, and Qualtrics. Political Sci. Res. Methods.

[61] Elamroussi A., Hanna J., Vera A. (2021). States begin scaling back daily Covid-19 data reporting as federal officials try to vaccinate more Americans. CNN.

[62] Bilandzic H., Kalch A., Soentgen J. (2017). Effects of goal framing and emotions on perceived threat and willingness to sacrifice for climate change. Sci. Commun..

[63] La Guardia J.G., Ryan R.M., Couchman C.E., Deci E.L. (2000). Within-person variation in security of attachment: A self-determination theory perspective on attachment, need fulfillment, and well-being. J. Personal. Soc. Psychol..

[64] Hayes A.F. Process for SPSS and SAS (Version 3.5.3). http://processmacro.org/download.html.

[65] Hayes A.F. (2018). Introduction to Mediation, Moderation, and Conditional Process Analysis: A Regression-Based Approach.

[66] Folkman S. (1984). Personal control and stress and coping processes: A theoretical analysis. J. Personal. Soc. Psychol..

[67] Lazarus R.S., Folkman S. (1984). Stress, Appraisal, and Coping.

[68] Whatley S.L., Foreman A.C., Richards S. (1998). The relationship of coping style to dysphoria, anxiety, and anger. Psychol. Rep..

[69] Genest M., Bowen R.C., Dudley J., Keegan D. (1990). Assessment of strategies for coping with anxiety: Preliminary investigations. J. Anxiety Disord..

[70] Reynolds-Tylus T., Lukacena K.M., Truban O. (2021). Message fatigue to bystander intervention messages: Examining pathways of resistance among college men. Health Commun..

[71] Hornik R.C., Hornik R.C. (2002). Public health communication: Making sense of contradictory evidence. Public Health Communication: Evidence for Behavior Change.

[72] Koh P.K.-K., Chan L.L., Tan E.-K. (2020). Messaging fatigue and desensitisation to information during pandemic. Arch. Med. Res..

[73] Dillard J.P., Nabi R.L. (2006). The persuasive influence of emotion in cancer prevention and detection messages. J. Commun..

[74] Staunton T.V., Alvaro E.M., Rosenberg B.D., Crano W.D. (2020). Controlling language and irony: Reducing threat and increasing positive message evaluations. Basic Appl. Soc. Psychol..

[75] Legault L., Gutsell J.N., Inzlicht M. (2011). Ironic effects of antiprejudice messages: How motivational interventions can reduce (but also increase) prejudice. Psychol. Sci..

[76] Motta M., Stecula D., Farhart C. (2020). How right-leaning media coverage of COVID-19 facilitated the spread of misinformation in the early stages of the pandemic in the U.S. Can. J. Political Sci..

[77] The Lancet Infectious D. (2020). The COVID-19 infodemic. Lancet Infect. Dis..

[78] Stroebe W., vanDellen M.R., Abakoumkin G., Lemay E.P., Schiavone W.M., Agostini M., Bélanger J.J., Gützkow B., Kreienkamp J., Reitsema A.M. (2021). Politicization of COVID-19 health-protective behaviors in the United States: Longitudinal and cross-national evidence. PLoS ONE.

[79] Halpern L.W. (2020). The politicization of COVID-19. AJN Am. J. Nurs..

[80] Gollust S.E., Nagler R.H., Fowler E.F. (2020). The emergence of COVID-19 in the US: A public health and political communication crisis. J. Health Politics Policy Law.

