# Supplementary material for: The Relationship between Future Anxiety Due to COVID-19 and Vigilance: The Role of Message Fatigue and Autonomy Satisfaction
Source: Int J Environ Res Public Health. 2022 Jan 18;19(3):1062. doi: 10.3390/ijerph19031062 (PMC8833904; doi:10.3390/ijerph19031062)
Supplement: Supplementary file 1 [file ijerph-19-01062-s001.zip › ijerph-1466623-supplementary.pdf]

Table S1.

Bivariate Correlations among Measured Variables

Variable	FAN	MFG	VIG	AUT	AGE	GEN	EDU	INC	RAC	POL	DTH	VAC	ERS
FAN													
MFG	.11*												
VIG	.15***	−.45***											
AUT	−.12**	.05	.15***										
AGE	−.19***	.01	−.10*	.08									
GEN	−.05	.03	.03	−.01	−.05								
EDU	.07	−.06	.10*	.003	−.09*	−.04							
INC	−.04	−.03	.16***	.11*	−.04	−.10*	.44***						
RAC	.01	−.11*	.12**	−.03	−.14**	−.01	−.004	−.01					
POL	.10*	−.36***	.32***	−.12**	−.21***	−.01	.07	−.03	.07				
DTH	.09*	−.04	.05	−.06	−.03	.05	.02	.03	.09*	.08			
VAC	.07	−.22***	.31***	−.03	.05	−.09*	.24***	.20***	.04	.23***	.09		
ERS	−.14**	.18***	−.28***	.03	.08	.09*	.03	.02	−.18***	−.09	−.04	−.09*	

$* p < .05$; $**p < .01$; $***p < .001$.

FAN = Future Anxiety

MFG = Message Fatigue

VIG = Vigilance

AUT = Autonomy Satisfaction

AGE = Age

GEN = Gender

EDU = Education

INC = Income

RAC = Race

POL = Political Orientation

DTH = COVID death

VAC = Vaccination

ERS = Eased Restrictions.
